# Analysis of the Genetic Diversity and Population Structure of Four Senegalese Sheep Breeds Using Medium-Density Single-Nucleotide Polymorphisms

**DOI:** 10.3390/ani12121512

**Published:** 2022-06-10

**Authors:** Ayao Missohou, Basse Kaboré, Laurence Flori, Simplice Bosco Ayssiwede, Jean-Luc Hornick, Marianne Raes, Jean-François Cabaraux

**Affiliations:** 1 Animal Production and Nutrition Unit, Inter-State School of Veterinary Science and Medicine (EISMV), Dakar BP 577, Senegal; bassekabore1@gmail.com (B.K.); ayissimbos@yahoo.fr (S.B.A.); 2 SELMET, INRAE, CIRAD, L’Institut Agro, University of Montpellier, 34000 Montpellier, France; laurence.flori@inrae.fr; 3 Department of Veterinary Management of Animal Resources, FARAH, Faculty of Veterinary Medicine, University of Liège, 4000 Liège, Belgium; jlhornick@uliege.be (J.-L.H.); jfcabaraux@uliege.be (J.-F.C.); 4 Department of Veterinary Medicine, URVI, University of Namur, 5000 Namur, Belgium; marianne.raes@unamur.be

**Keywords:** SNP, sheep, Senegal

## Abstract

**Simple Summary:**

This paper reported genetic parameters of four Senegalese sheep breeds, in relation to inbreeding, diversity and genetic proximity. The results provide informations on genetic conservation and adaptability of the breeds in the Senegalese context.

**Abstract:**

In Senegal, sheep breeds have adapted to their environment and play a key socio-economic role. This study aimed to explore the genetic diversity and structure of four Senegalese sheep breeds (Peul-peul, Djallonke, Touabire, and Ladoum) and their relationships with global sheep breeds. To that end, forty-seven sheep were genotyped using the OvineSNP50 BeadChip, and these genotypic data were analysed with those of 73 sheep breeds representative of worldwide ovine diversity (2729 animals). The average observed heterozygosity (Ho) ranged from 0.293 in Djallonke sheep to 0.339 in Touabire sheep. The estimated Fis values were low, ranging from 0.019 for Ladoum to 0.034 for Peul-peul sheep. The estimated Fst values were low (0.003–0.044) among the trypanosusceptible breeds (Peul-peul, Touabire, and Ladoum) but high between the previous breeds and the trypanotolerant Djallonke breed (0.075–0.116), indicating better genetic conservation of the Djallonke sheep. A principal component analysis revealed clustering of the Senegalese sheep breeds according to their geographic distribution. However, owing to genetic improvement practices, the introgression of Touabire sheep blood seems to have reshaped the genetic landscape of the trypanosusceptible sheep breeds in Senegal. The Senegalese sheep breeds showed lower genetic diversity than their presumed ancestral sheep breeds of the Middle East. They also presented some relatedness with Caribbean sheep breeds, which reveals their contribution to the global genetic diversity and to the development of Caribbean sheep breeds.

## 1. Introduction

Sheep, the most commonly farmed species after poultry, play a crucial socio-economic role in Senegal. In addition to their economic value, sheep are the preferred meat among Senegalese consumers [1] and the primary animal species offered as sacrifice during various social (weddings) and religious (baptisms and mainly Tabaski, also referred to as Aïd el Kabir) events. Hence, the demand for sheep in Senegal continues to increase (810,000 heads sacrificed in 2020, according to official estimations) and is largely covered by imports of live sheep from neighbouring countries (Mauritania and Mali) [2]. The demand for sheep is not just quantitative; in large cities such as Dakar, it is also qualitative, especially on the occasion of Tabaski, during which sheep have a representative function and are a status symbol for their owner [3].

The sheep breeds traditionally raised in Senegal include Peul-peul and Djallonke, which are thin-tailed and domesticated in the central Fertile Crescent. They are thought to descend from the Asiatic mouflon (Ovis orientalis) [4]. According to archaeozoological data, they were introduced to Africa via the Isthmus of Suez and/or the Southern Sinai Peninsula between 7500 and 7000 BP and reached West Africa by 3700 BP [5]. Peul-peul is a long-legged sheep breed (65–75 cm tall) and is related to the Sahelian sheep found in the arid and semi-arid zones of the Sahel belt in West Africa. They are considered the most ancient sheep on the continent, and their convex nose, pendulous ears, and exceptionally long legs closely resemble those of sheep found in ancient Egypt [6,7]. Djallonke sheep are related to tropical dwarf trypanotolerant sheep found in sub-humid and humid zones of West and Central Africa and are smaller (40–60 cm) than Peul-peul sheep. Both breeds are reared under extensive low-input production systems where animals graze natural pastures all year round and certain supplements (agricultural by-products) are sometimes fed [8]. These native Senegalese sheep breeds are well known for their hardiness, i.e., they can live on poor-quality feed and under high temperatures and disease pressure. However, their productivity is low––their mature body weights are 30–45 kg for Peul-peul and 20–30 kg for Djallonke sheep. The search for good-quality sheep has led to the increased introduction of different sheep breeds to Senegal for genetic improvement purposes [9]. Since the introduction of European and Moroccan breeds is considered a failure, the Mauritanian short-haired Touabire sheep, known as one of the largest breeds in the sub-region, has been widely used in Senegal [8], and its cross with Peul-peul sheep is named Waralé [9]. To deal with the high demand for these aesthetic-judged sheep, urban and peri-urban breeders simultaneously initiated a breeding program that yielded a new breed called Ladoum. According to Ousseini [10], the Ladoum breed could have been developed in Thiès from the nucleus of Touabire sheep imported from Mauritania in the 1970s or from a local breed through selection. From Thiès, it extended to other big Senegalese cities, mainly Dakar, which is its main basin. These sheep are extremely popular among the Senegalese for their large size and social-based aesthetic criteria and are used for crossbreeding purposes. Such genetic improvement practices threaten native Senegalese sheep breeds, the hardiness of which is the cornerstone of sheep farming systems in Senegal. This could deteriorate the already precarious conditions of animal genetic resources in West Africa, where under the combined effects of climate change, drought, and increase in the amplitude of transhumance, there is an increasing erosion of genetic diversity. The erosion of locally adapted genetic resources will significantly limit the options and capacity to cope with changes in production environments and breeding goals [11]. 

The genetic characterisation of these local breeds is an essential step toward providing guidelines for future conservation and improving the management of sheep genetic diversity. Early studies on Senegalese sheep breeds used morphometric traits [9,12] or blood markers [13]. However, in recent years, analyses using single-nucleotide polymorphisms (SNPs) have become the standard approach for genetic diversity analysis and genome-wide studies [11]. The recently developed genome-wide medium-density ovine SNP array is an excellent tool for investigating genetic diversity at high resolutions, for inferring population history, and for mapping genomic regions subject to selection and adaptation [14,15,16]. Therefore, it offers the opportunity to trace historical patterns of Senegalese sheep breed structures on a broader geographic scale.

Here, we aim to explore the genetic diversity, population structure, and relationships among the main Senegalese sheep breeds—the two local breeds Djallonke and Peul-peul, the newly developed breed Ladoum, and Touabire sheep imported from Mauritania—using a newly developed genotyping dataset. We analysed 47 unrelated Senegalese sheep by genotyping using OvineSNP50 BeadChip from Illumina and combined these genotypes with those of 73 other sheep populations representative of the sheep genetic diversity.

## 2. Material and Methods

### 2.1. Breeds, Sampling Strategy, and DNA Extraction

Four sheep breeds were sampled in this study: Djallonke (DJA), Peul-peul (PEU), Touabire (TOU), and Ladoum (LAD). In Senegal, Djallonke sheep are primarily found in Casamance. The Kolda Region, where the Animal Production Research Centre of Kolda hosts technical facilities for blood sampling (n = 12), was the focus of our study. Peul-peul sheep are found in tsetse fly-free zones of rural areas. For this breed, blood sampling (n = 12) was conducted in Dahra in the Djolof, known as the breed birthplace. This blood sampling was organised by the Animal Production Research Centre of Dahra (CRZ-D). One blood sample was collected per village, with a minimum distance of 12 km between two villages, except for the Ladoum breed, due to its in-city geographical dispersion. Ladoum blood samples (n = 12) were collected in the Dakar Region from farms whose owners were members of the ADAM, a farmer association specialised in the breeding of Ladoum sheep. For these three breeds, the farms and the animals in each farm were randomly chosen. Although Touabire sheep are found in the Northern region of Senegal [17], for breed purity reasons, samples (n = 12) were collected among sheep imported from Mauritania (birthplace of the Touabire breed) available for sale at a livestock market in Dakar. DNA was extracted from 5 mL of frozen blood, as described by Jeanpierre [18], at the Molecular Biology Laboratory of the Inter-State School of Veterinary Science and Medicine of Dakar.

### 2.2. DNA Genotyping

The DNA samples were genotyped using OvineSNP50 BeadChip from Illumina (www.illumina.com, accessed on 30 March 2022) at the GIGA research center (University of Liège, Belgium). The genotypes were then integrated into the WIDDE database (www.widde.toulouse.inrae.fr, accessed on 30 March 2022) [19]. The quality filtering was performed using WIDDE utilities. In particular, SNPs genotyped for <75% of individuals in at least one breed (minimal individual genotyping call rate was set to 90% throughout) and animals genotyped for <95% of the SNPs were discarded. SNPs with a minor allele frequency of <1% and those deflecting from the Hardy–Weinberg equilibrium (exact test *p*-value <0.001) were also filtered out. Two genotyping datasets were extracted from WIDDE for further analyses, i.e., the SEN-Set and the WORLD-Set (Table 1). Finally, the SEN-Set included 47 animals from the four Senegalese breeds (a Djallonke sample was excluded) genotyped for 44,321 SNPs after discarding 2498 SNPs. The WORLD-Set consisted of 2776 animals belonging to 77 sheep breeds, including the four Senegalese breeds and 73 breeds representative of the worldwide ovine diversity [20,21] genotyped for 40,916 SNPs (5903 SNPs excluded).

### 2.3. Analyses of Sheep Breed Genetic Diversity and Structure 

A principal component analysis was performed using smartpca software [23] and visualised with R package ade4 [24]. Unsupervised genotype-based hierarchical clustering of the individual samples was performed using the maximum-likelihood method implemented in Admixture 1.06 [25]. 

Observed heterozygosities, expected heterozygosities [26], and F-statistics (within a population Fis and between populations Fst) [27] were estimated using custom R functions.

For Senegalese sheep breeds, the gene flow parameter Nm was calculated using the following formula [28]:

Nm = 1 − Fst/4 Fst.(1)

ASDs (allele sharing distances) were computed for each pair of individuals using all available SNP information. For a given pair of individuals i and j,

ASD = 1 − xij,(2)

where xij represents the proportion of alleles alike in state, averaged over all genotyped SNPs. A neighbour-joining (NJ) tree was computed based on the ASD matrix using R package ape software [29].

## 3. Results and Discussion

### 3.1. Within-Breed Genetic Diversity 

The genetic diversity of the Senegalese sheep breeds explored using the SEN-Set is presented in Table 2. The observed heterozygosity (Ho) varied from 0.302 in the Djallonke sheep to 0.323 in the Ladoum sheep and from 0.344 in the Peul-peul sheep to 0.349 in the Touabire sheep. The expected heterozygosity (He) was the lowest in the Djallonke sheep and highest in the Touabire sheep. These estimates of within-breed genetic diversity were in the range of previously published data based on SNPs [20,30,31,32,33] but were low compared to those reported for similar breeds in West Africa, for which Ho ranged from 0.583 to 0.703 [34,35,36]. However, the latter studies were based on a low number of microsatellite polymorphisms (n = 12–27). The lowest genetic diversity observed in the Djallonke sheep could have resulted from the cordon sanitaire around this breed, created by tsetse flies (i.e., vectors of the Animal African Trypanosomosis) present within its habitat, which limited the introduction of trypanosusceptible Senegalese sheep breeds for crossbreeding purposes. The estimated Fis values were low (ranging from 0.019 for Ladoum to 0.034 for Peul-peul Table 2) and in the range of the Fis of other sheep breeds for which weak-to-moderate inbreeding coefficients were reported [20,30,31].

### 3.2. Population Relationships and Structure

Population relationships among the Senegalese breeds were determined by calculating a pairwise Fst and gene flow parameter Nm (assessed based on the number of migrants per generation) matrix (Table 3). 

The estimated Fst values among the trypanosusceptible breeds Peul-peul, Touabire, and Ladoum were low (0.0033–0.0438) and in the range of previously reported results [11,37]. The largest computed values were found between the Djallonke and Ladoum sheep breeds (0.1156), between the Djallonke and Peul-peul sheep breeds (0.0772), and between the Djallonke and Touabire sheep breeds (0.0749) and could reflect the effectiveness of sanitary barriers against the introgression of other breeds imposed by trypanosomosis, as stated previously. Moreover, in the absence of artificial insemination technology in sheep in Senegal, the small size of the Djallonke sheep could not support crossbreeding with large breeds such as Ladoum and Touabire. We did not observe in Senegalese sheep the trends reported in cattle of increased introgression of the trypanosusceptible breed into trypanotolerant cattle [38,39,40]. The largest Nms were found between the Peul-peul and Touabire sheep (73.3) and between the Touabire and Ladoum sheep (8.7). Apart from the husbandry system, this could have resulted from the common use of Touabire sheep by Senegalese farmers to upgrade the size of the Peul-peul sheep for a better sale price during Tabaski. These crossbreeding practices were also enforced by policy makers in CRZ-D who implemented the nucleus of Touabire sheep [8]. Moreover, the Dahra region, from where the Peul-peul sheep samples were collected, houses the biggest livestock market of Senegal [41]. It is connected to various markets via many roads, including those from Mauritania, and these could serve as crossbreeding paths between the Peul-peul sheep and other sheep breeds. The second highest Nm (8.7) was observed between the Touabire and Ladoum sheep, supporting the hypothesis related to the Touabire origin of the Ladoum sheep.

**Figure 1:**
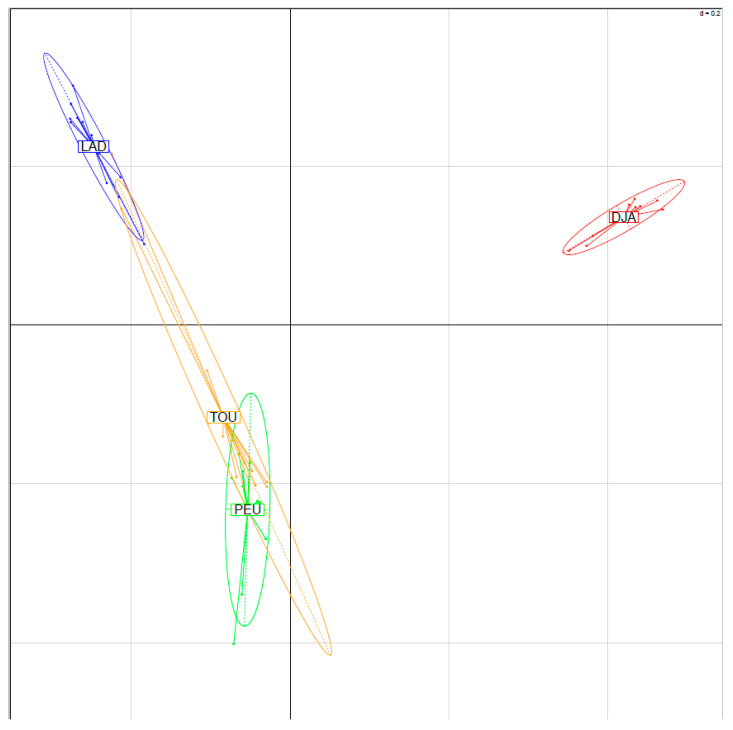
PCA results obtained with the 47 individuals of the four Senegalese breeds genotyped for 44,321 SNPs (SEN-Set). Individuals are plotted according to their coordinates on the first two principal components. Ellipses characterise the dispersion of each breed around its centre of gravity.

The relationships within individuals and among Senegalese sheep breeds, as illustrated by the principal component analysis, are shown in Figure 1. The variations accounted for by the first and the second principal components were lower (6.23% and 4.23%, respectively) than the variations reported by Edea et al. [11] in Ethiopian sheep breeds and by Sandenberg et al. [42] in South African sheep breeds; however, our findings corroborated those of Kijas et al. [20] and Deniskova et al. [37]. According to Kijas et al. [20], the 20 largest principal components accounted for only 16% of the total variation. The principal component analysis clustered the breeds into two main groups, i.e., the trypanotolerant Djallonke sheep and the trypanosusceptible Peul-peul, Touabire, and Ladoum sheep. Currently, West African small ruminants are clustered in the Sahelian type (with trypanosusceptible breeds) and in the forest or savannah type (with the trypanotolerant Djallonke sheep) [43,44]. Thus, we confirmed the findings of many previous studies indicating sheep breed clustering according to their geographic distribution [11,20].

**Figure 2:**
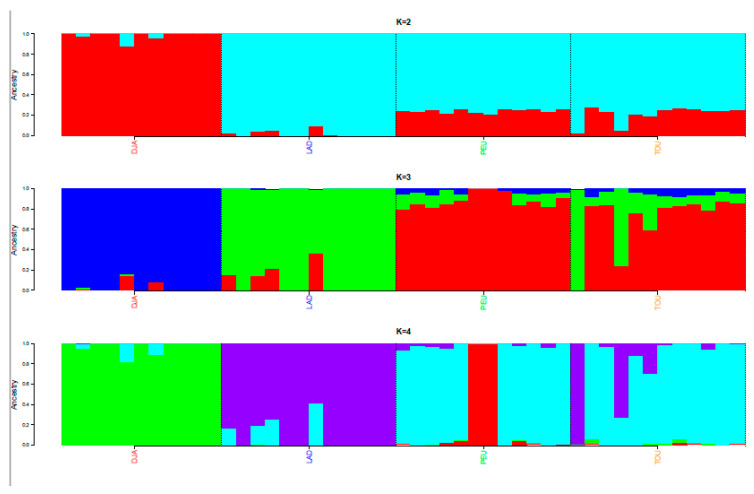
Unsupervised hierarchical clustering of the 47 individuals of the four Senegalese breeds genotyped for 44,321 SNPs (SEN-Set). Results for an inferred number of clusters k varying from 2 to 4 are shown.

The Admixture analysis (Figure 2) provided additional evidence of the distinctness of the Djallonke sheep that clustered in a separate group at k = 2. At k = 3, the clear contribution of the Touabire breed to the other trypanosusceptible breeds (i.e., Ladoum and Peul-peul) was illustrated and was consistent with the results of the principal component analysis. 

**Figure 3:**
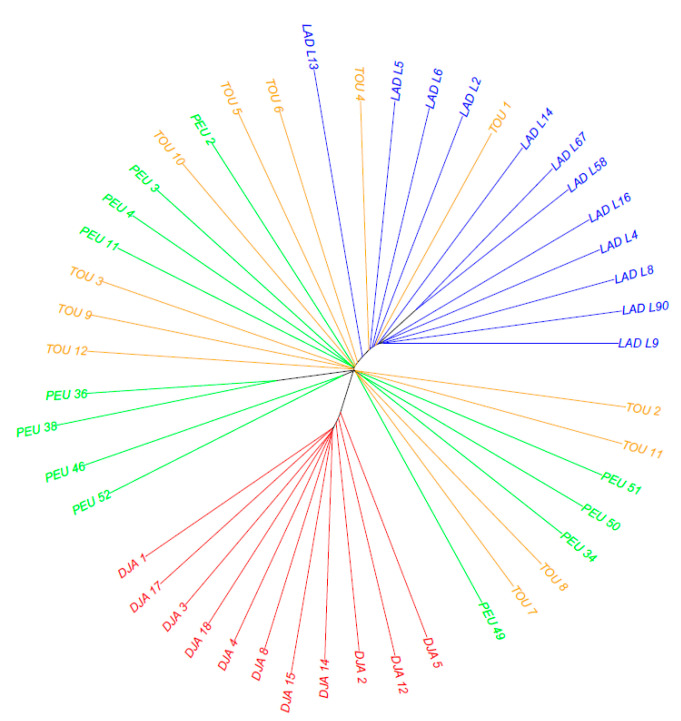
Neighbour-joining tree relating the 47 individuals of the four Senegalese breeds. The tree was constructed using allele sharing distances averaged over 44,321 SNPs. Labels are coloured according to the individual breed of origin.

The NJ tree constructed using ASD genetic distance (Figure 3) confirmed the clustering of the Senegalese sheep breeds into three clads: a clad with the Djallonke sheep; another one with the Ladoum sheep and some Touabire sheep; and finally, one with a mixture of the Peul-peul and Touabire sheep. Consistent with the findings of Ciani et al. [21], we observed genetic sub-structuring in the Senegalese Sahelian-type breeds, which reflects recent herd management and trade practices wherein the Ladoum sheep emerge at the expense of the Peul-peul and Touabire sheep.

### 3.3. Senegalese Sheep Breeds in the Global Context

**Figure 4:**
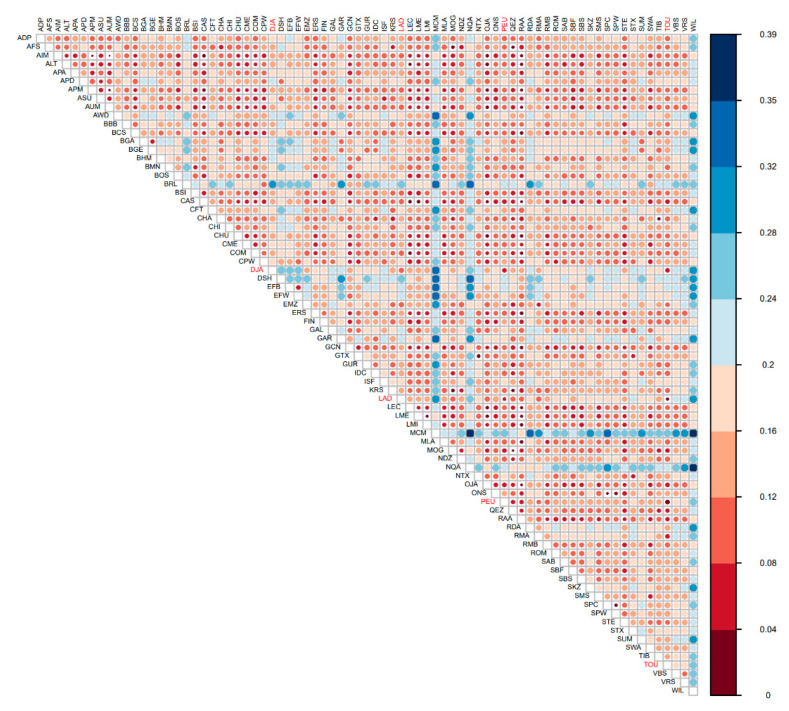
Pairwise Fst among 77 breeds estimated from different SNP datasets. Acronyms of Senegalese sheep breeds are coloured in red.

To compare genetic diversity and trace historical patterns of Senegalese sheep breeds structure on a broader geographic scale, the genotypic data of the WORLD-Set were analysed. The Ho and He were in the range of genetic diversity computed for other African breeds (African Red Dorper, Ronderib Africkaner, Red Masai, and Ethiopian), but were slightly lower than those computed for their presumed ancestral breeds from South-East Asia (Afshari, Karakas, Moghani, Norduz, and Quel) (Table 4) and aligned with the known decline in genetic diversity with the distance separating location from the domestication centre [45,46]. The pairwise Fst values (Figure 4) were low between Senegalese sheep breeds (mainly Peul-peul and Touabire) and some Middle Eastern sheep breeds (i.e., Qezel and Moghani) and Spanish sheep breeds (i.e., Rasa Aragonesa and Castellana). They were the highest with MacArthur merino and some breeds from the UK (i.e., Wiltshire, Border Leicester, and Dorset Horn).

**Figure 5:**
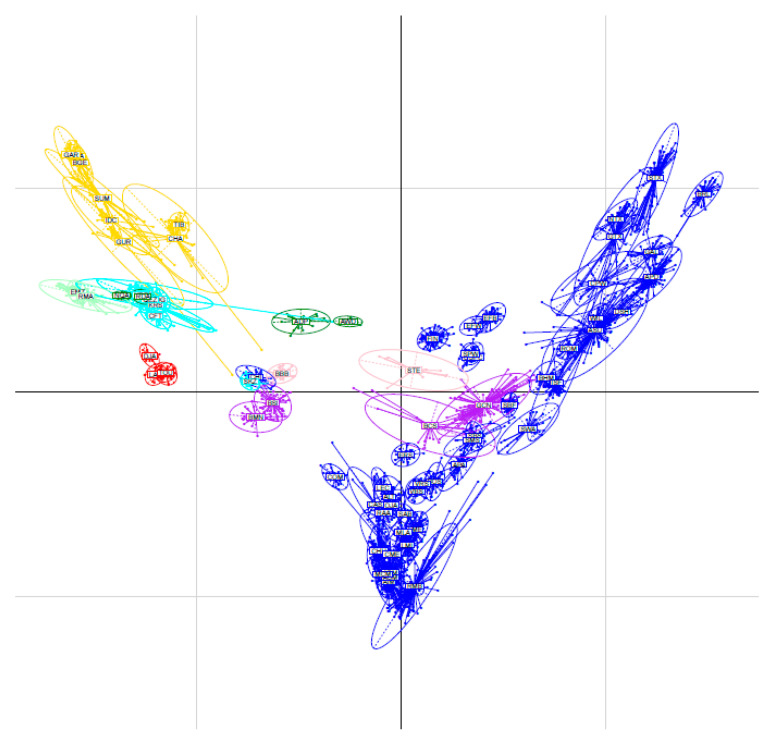
Results of the principal component analysis (PCA) obtained using genotyping data from the WORLD-set (2776 individuals from 77 populations including the 4 Senegalese sheep breeds, genotyped for 40,916 SNPs). The individuals are plotted on the first two principal components according to their coordinates. Ellipses characterise the dispersion of each population around the centre of gravity. Populations are coloured according to their geographic origin (i.e., in blue for Europe, in purple for America, in pink for the Caribbean, in yellow for Asia, in cyan for the Middle East, in light green for East Africa, and in dark green for South Africa).

The principal component analysis performed using the 77 breeds is presented in Figure 5, with PC1 and PC2 accounting for only 2.73% and 1.57%, respectively, of the total variation. Senegalese sheep breeds formed a uniform group that clustered separately from the other breeds. However, they are close to East African breeds (Ethiopian Menz and Red Masai) and to Southern African breeds (Namaqua Africaner and Ronderib Africaner). The relatedness of the African breeds was in accordance with their introduction throughout the Horn of Africa, from where they spread northwards (alongside the Mediterranean Sea) to West Africa and southwards to Eastern and Southern Africa via Ethiopia and Kenya [47]. The Senegalese sheep breeds are also similar to the Middle Eastern sheep breeds from Iran (Afshari, Mohghani, and Qezel), Cyprus (Cyprus Fat tail), and Turkey (Karakas, Norduz, and Sakiz) as well as to the Greek breed Chios.

The proximity between Senegalese sheep breeds and some Brazilian breeds (i.e., Morado Nova and Santa Ines) and a Caribbean hair sheep breed (i.e., Barbados Black Belly) also highlighted by the principal component analysis could be supported by colonisation patterns. According to Spangler et al. [32], the birth places of Caribbean sheep breeds were the regions of highest slave importation and, by correlation, of West African sheep breeds. Thus, our results are consistent with a West African sheep heritage of Caribbean hair sheep, as proposed by Spangler et al. [32], which may have contributed to their disease resistance and economic viability.

**Figure 6:**
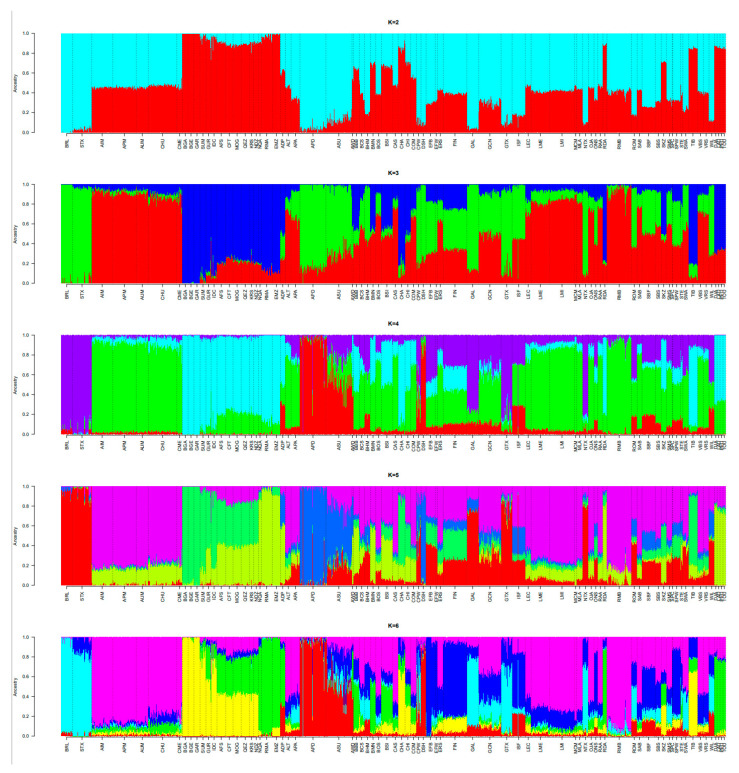

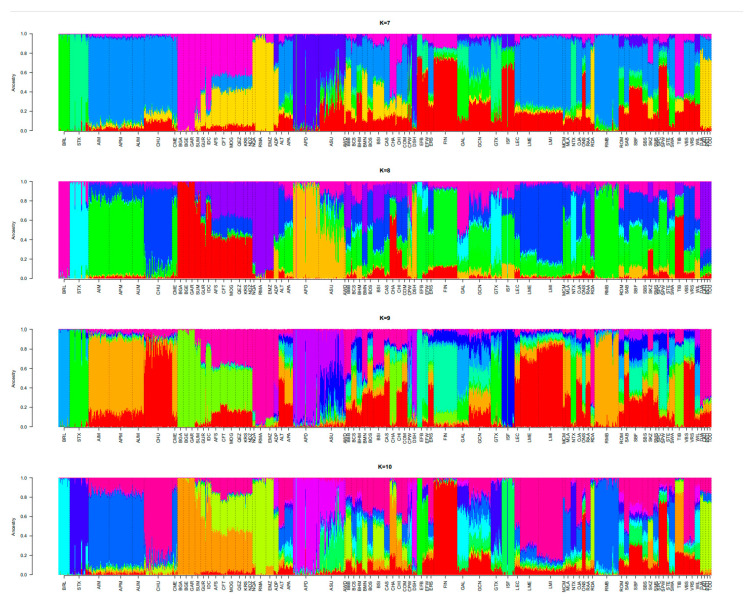
Unsupervised hierarchical clustering of the 2776 individuals of 77 breeds genotyped for 40,916 SNPs (WORLD-Set). Results for an inferred number of clusters k varying from 2 to 10 are shown. Each breed ID is coloured according to the breed geographic origin (i.e., in blue for Europe, in purple for America, in pink for the Caribbean, in yellow for Asia, in cyan for the Middle East, in light green for East Africa, and in dark green for South Africa).

The results of the admixture analysis for k = 2–10 (Figure 6) confirmed our previous results and additionally suggested the relatedness of Senegalese sheep breeds primarily with most of the presumed ancestral breeds of the Middle East. 

## 4. Conclusions

In summary, we observed a low genetic diversity among the trypanosusceptible Senegalese sheep breeds, whereas the Djallonke sheep clearly deflected from the other breeds. The increased use of the Touabire sheep to upgrade the native Senegalese breeds seems to have led to the dilution of the Peul-peul breed and allowed for the emergence of the Ladoum breed. Even if the Djallonke sheep seemed to have a better conservation status in relation to the sanitary barriers imposed by trypanosomiasis to the introgression of other breeds, this should be a matter of concern that appeals to the enforcement of a regulatory framework for breed conservation and genetic improvement in Senegal. This study additionally emphasizes the contribution of Senegalese sheep breeds to the global genetic diversity and especially to the development of Caribbean hair sheep breeds.

## Figures and Tables

**Table 1:** List of the sheep breeds of the WORLD-Set considered in the current study.

Breed ID	Breed Name	Location	Area of Origin	Nb	Reference
ADP	African Dorper	South Africa	Souh Africa	21	[20]
AFS	Afshari	Iran	Middle East	37	[20]
AIM	Australian Industry Merino	Australia	Europe	88	[20]
ALT	Altamurana	South Italia	Europe	24	[20]
APA	Arapawa	New Zealand	Europe	37	[20,22]
APD	Australian Poll Dorset	Australia	Europe	108	[20]
APM	Australian Poll Merino	Australia	Europe	98	[20]
ASU	Australian Suffolk	Australia	Europe	109	[20]
AUM	Australian Merino	Australia	Europe	50	[20]
AWD	African White Dorper	South Africa	South Africa	6	[20]
BBB	Barbados Black Belly	Barbados	Caribbean	24	[20]
BCS	Brazilian Creole	Brazil	America	23	[20]
BGA	Bangladeshi Garole	Bangladesh	Asia	24	[20]
BGE	Bangladeshi East BGE	Bangladesh	Asia	24	[20]
BHM	Black Headed Mountain	UK	Europe	24	[20]
BMN	Morada Nova	Brazil	America	22	[20]
BOS	Bundner Oberlander Sheep	Germany	Europe	24	[20]
BRL	Border Leicester	UK	Europe	48	[20]
BSI	Santa Ines	Brazil	America	47	[20]
CAS	Castellana	Spain	Europe	23	[20]
CFT	Cyprus Fat Tail	Cyprus	Middle East	30	[20]
CHA	Changthangi	Indian	Asia	29	[20]
CHI	Chios	Greece	Europe	23	[20]
CHU	Churra	Spain	Europe	120	[20]
CME	Chinese Merino	China	Europe	23	[20]
COM	Comisana	Italia	Europe	24	[20]
CPW	Australian Coopworth	Australia	Europe	19	[20]
DJA	Djallonke	Senegal	West Africa	11	this study
DSH	Dorset Horn	UK	Europe	21	[20]
EFB	East Friesian Brown	Germany	Europe	39	[20]
EFW	East Friesian White	Germany	Europe	9	[20]
EMZ	Ethiopian Menz	Ethiopia	East Africa	34	[20]
ERS	Engadine Red Sheep	Swiss	Europe	24	[20]
FIN	Finnsheep	Finland	Europe	99	[20]
GAL	Galway	UK	Europe	49	[20]
GAR	Indian Garole	India	Asia	26	[20]
GCN	Gulf Coast Native	US	America	94	[20]
GTX	German Texel	Germany	Europe	46	[20]
GUR	Garut	Indonesia	Asia	22	[20]
IDC	Deccani	India	Asia	24	[20]
ISF	Irish Suffolk	UK	Europe	55	[20]
KRS	Karakas	Turkey	Middle East	18	[20]
LAD	Ladoum	Senegal	West Africa	12	this study
LEC	Leccese	Italia	Europe	24	[20]
LME	Lacaune (meat)	France	Europe	78	[20]
LMI	Lacaune (milk)	France	Europe	103	[20]
MCM	Macarthur Merino	Australia	Europe	10	[20]
MLA	Merinolandschaf	Germany	Europe	24	[20]
MOG	Moghani	Iran	Middle East	34	[20]
NDZ	Norduz	Turkey	Middle East	20	[20]
NQA	Namaqua Afrikaner	South Africa	South Africa	12	[20]
NTX	New Zealand Texel	New Zealand	Europe	24	[20]
OJA	Ojalada	Spain	Europe	24	[20]
ONS	Old Norwegian Spaelsau	Norway	Europe	15	[20]
PEU	Peul	Senegal	West Africa	12	this study
QEZ	Qezel	Iran	Middle East	35	[20]
RAA	Rasa Aragonesa	Spain	Europe	22	[20]
RDA	Ronderib Afrikaner	South Africa	South Africa	17	[20]
RMA	Red Maasai	Kenya	East Africa	45	[20]
RMB	Merinos de Rambouillet	France	Europe	102	[20]
ROM	New Zealand Romney	New Zealand	Europe	24	[20]
SAB	Sardinian Ancestral Black	Italia	Europe	20	[20]
SBF	Scottish Blackface	UK	Europe	56	[20]
SBS	Swiss Black	Swiss	Europe	24	[20]
SKZ	Sakiz	Turkey	Middle East	22	[20]
SMS	Swiss Mirror	Swiss	Europe	24	[20]
SPC	Spael	Norway	Europe	3	[20]
SPW	Spael	Norway	Europe	32	[20]
STE	St Elizabeth	Jamaica	Caribbean	10	[20]
STX	ScottishTexel	UK	Europe	80	[20]
SUM	Sumatra	Indonesia	Asia	24	[20]
SWA	Swiss White Alpine	Switzerland	Europe	24	[20]
TIB	Tibetan	China	Asia	37	[20]
TOU	Touabire	Senegal	West Africa	12	this study
VBS	Valais Blacknose	Canada	Europe	24	[20]
VRS	Valais Red	Switzerland	Europe	24	[20]
WIL	Wiltshire	UK	Europe	23	[20]

Nb: Number of animals after filtering.

**Table 2:** Genetic diversity estimates in the four studied Senegalese sheep breeds.

Breed	Nb	Ho	He	Fis
Djallonke	11	0.302	0.286	0.024
Peul-peul	12	0.344	0.322	0.034
Touabire	12	0.349	0.330	0.024
Ladoum	12	0.323	0.307	0.019

Nb: Number of animals after filtering.

**Table 3:** Pairwise genetic differentiation (Fst, lower triangular matrix) and gene flow (Nm, upper triangular matrix) among the Senegalese sheep breeds based on the polymorphisms of 44,321 SNPs.

	Djallonke	Ladoum	Peul-Peul	Touabire
Djallonke	-	1.913	2.980	3.079
Ladoum	0.116	-	5.432	8.679
Peul-peul	0.077	0.044	-	73.279
Touabire	0.075	0.028	0.003	-

**Table 4:** Within-breed genetic diversity indices in 77 breeds estimated from different SNP datasets.

Breed ID	Breed Name	Nb	Ho	He	Fis
ADP	African Dorper	21	0.342	0.337	−0.012
AFS	Afshari	37	0.355	0.348	−0.020
AIM	Australian Industry Merino	88	0.367	0.374	0.019
ALT	Altamurana	24	0.360	0.369	0.026
APA	Arapawa	37	0.326	0.349	0.067
APD	Australian Poll Dorset	108	0.345	0.344	−0.003
APM	Australian Poll Merino	98	0.372	0.375	0.008
ASU	Australian Suffolk	109	0.372	0.372	−0.001
AUM	Australian Merino	50	0.363	0.374	0.031
AWD	African White Dorper	6	0.327	0.301	−0.086
BBB	Barbados Black Belly	24	0.317	0.341	0.070
BCS	Brazilian Creole	23	0.329	0.371	0.114
BGA	Bangladeshi Garole	24	0.286	0.307	0.069
BGE	Bangladeshi East BGE	24	0.270	0.313	0.140
BHM	Black Headed Mountain	24	0.330	0.340	0.029
BMN	Morada Nova	22	0.310	0.319	0.028
BOS	Bundner Oberlander Sheep	24	0.359	0.347	−0.034
BRL	Border Leicester	48	0.290	0.291	0.001
BSI	SantaInes	47	0.345	0.353	0.023
CAS	Castellana	23	0.378	0.375	−0.008
CFT	Cyprus Fat Tail	30	0.326	0.315	−0.036
CHA	Changthangi	29	0.332	0.353	0.060
CHI	Chios	23	0.324	0.330	0.019
CHU	Churra	120	0.359	0.362	0.008
CME	Chinese Merino	23	0.365	0.359	−0.017
COM	Comisana	24	0.372	0.368	−0.009
CPW	Australian Coopworth	19	0.378	0.366	−0.033
DJA	Djallonke	11	0.293	0.300	0.024
DSH	Dorset Horn	21	0.315	0.296	−0.065
EFB	East Friesian Brown	39	0.295	0.300	0.018
EFW	East Friesian White	9	0.316	0.310	−0.018
EMZ	Ethiopian Menz	34	0.321	0.325	0.013
ERS	Engadine Red Sheep	24	0.369	0.366	−0.009
FIN	Finnsheep	99	0.345	0.356	0.031
GAL	Galway	49	0.335	0.334	−0.003
GAR	Indian Garole	26	0.288	0.295	0.024
GCN	Gulf Coast Native	94	0.364	0.377	0.034
GTX	German Texel	46	0.349	0.354	0.016
GUR	Garut	22	0.333	0.332	−0.003
IDC	Deccani	24	0.335	0.337	0.005
ISF	Irish Suffolk	55	0.315	0.330	0.046
KRS	Karakas	18	0.355	0.347	−0.022
LAD	Ladoum	12	0.313	0.319	0.019
LEC	Leccese	24	0.346	0.372	0.070
LME	Lacaune (meat)	78	0.365	0.371	0.016
LMI	Lacaune (milk)	103	0.361	0.365	0.009
MCM	Macarthur Merino	10	0.231	0.229	−0.011
MLA	Merinolandschaf	24	0.362	0.359	−0.007
MOG	Moghani	34	0.363	0.363	−0.001
NDZ	Norduz	20	0.352	0.337	−0.046
NQA	Namaqua Afrikaner	12	0.287	0.252	−0.140
NTX	NewZealand Texel	24	0.344	0.347	0.008
OJA	Ojalada	24	0.376	0.377	0.001
ONS	Old Norwegianspaelsau	15	0.339	0.358	0.054
PEU	Peul	12	0.333	0.345	0.035
QEZ	Qezel	35	0.361	0.366	0.015
RAA	Rasaaragonesa	22	0.384	0.383	−0.002
RDA	Ronderib Afrikaner	17	0.315	0.300	−0.048
RMA	Red Maasai	45	0.324	0.323	−0.004
RMB	Merinos de Rambouillet	102	0.345	0.357	0.032
ROM	New Zealand Romney	24	0.347	0.355	0.023
SAB	SardDSHinian Ancestral Black	20	0.355	0.339	−0.049
SBF	Scottish Blackface	56	0.361	0.360	−0.002
SBS	Swiss Black	24	0.354	0.352	−0.006
SKZ	Sakiz	22	0.333	0.313	−0.065
SMS	Swiss Mirror	24	0.356	0.350	−0.015
SPC	Spael	3	0.334	0.335	0.001
SPW	Spael	32	0.330	0.334	0.012
STE	St Elizabeth	10	0.373	0.371	−0.005
STX	ScottishTexel	80	0.349	0.331	−0.053
SUM	Sumatra	24	0.303	0.315	0.037
SWA	Swiss White Alpine	24	0.354	0.351	−0.008
TIB	Tibetan	37	0.323	0.341	0.052
TOU	Touabire	12	0.339	0.347	0.025
VBS	Valais Blacknose	24	0.303	0.318	0.049
VRS	Valais Red	24	0.323	0.314	−0.031
WIL	Wiltshire	23	0.269	0.267	−0.008

Nb: Number of animals after filtering, Ho: observed heterozygosity, He: expected heterozygosity, Fis: F-statistics within a population. Color fonts refer to breeds of the current study.

## References

[1] Seydi M., Ba Y.M. (1992). Les viandes rechercheés par les Sénégalais. Viandes Et Prod. Carnés.

[2] Ninot O. (2010). Des moutons pour la fête: L’approvisionnement de Dakar en moutons de Tabaski. Les Cah. D’outre-Mer.

[3] Brisebarre A.M. (2003). La Fête de la Tabaski en Milieu Urbain au Sénégal. Enjeux Culturels, Sociaux et Économiques; Rapport de Recherche; Conseil pour le Développement de la Recherche en Sciences Sociales en Afrique (CODESRIA).

[4] Bruford M.W., Townsend S.J., Zeder M.A., Bradley D.G., Emshwiller E., Smith B.D. (2006). Mitochondridal DNA diversity in modern sheep. Documenting Domestication: New Genetic and Archaeological Paradigms.

[5] Close A., Klees F., Kuper R. (1992). Holocene occupation of the eastern Sahara. New Light on the Northeast African Past.

[6] Ryder M.L. (1983). Sheep and Man.

[7] Ryder M.L., Mason I.L. (1984). Sheep. Evolution of Domesticated Animals.

[8] Fall A. (2000). Peul, Touabire and Djallonke sheep breeding programmes in Senegal. Proceedings of the Workshop on Developing Breeding Strategies for Lower Input Animal Production Environments.

[9] Ndiaye B., Diouf M.N., Ciss M., Wane M., Diop M., Sembène M. (2018). Morphologie et pratiques d’élevage du mouton peul-peul du sénégal. Int. J. Adv. Res..

[10] Ousseini H. (2011). Analyse Socioéconomique des Élevages du Mouton Ladoum Dans la Commune de Thiès/Sénégal. Master’s Thesis.

[11] Edea Z., Dessie T., Dadi H., Do K.-T., Kim K.-S. (2017). Genetic Diversity and Population Structure of Ethiopian Sheep Populations Revealed by High-Density SNP Markers. Front. Genet..

[12] Gueye A. (1997). Moutons et Chèvres du Sénégal: Caractérisation Morphobiométrique et Typage Sanguin. Ph.D. Thesis.

[13] Missohou A., Nguyen T.C., Sow R., Gueye A. (1999). Blood Polymorphism in West African Breeds of Sheep. Trop. Anim. Health Prod..

[14] Kijas J.W., Townley D., Dalrymple B.P., Heaton M.P., Maddox J.F., McGrath A., Wilson P., Ingersoll R.G., McCulloch R., McWilliam S. (2009). A Genome Wide Survey of SNP Variation Reveals the Genetic Structure of Sheep Breeds. PLoS ONE.

[15] Yang J., Li W.-R., Lv F.-H., He S.-G., Tian S.-L., Peng W.-F., Sun Y.-W., Zhao Y.-X., Tu X.-L., Zhang M. (2016). Whole-Genome Sequencing of Native Sheep Provides Insights into Rapid Adaptations to Extreme Environments. Mol. Biol. Evol..

[16] Zhao Y.X., Yang J., Lv F.H., Hu X.J., Xie X.L., Zhang M., Li W.R., Liu M.J., Wang Y.T., Li J.Q. (2017). Genomic Reconstruction of the History of Native Sheep Reveals the Peopling Patterns of Nomads and the Expansion of Early Pastoralism in East Asia. Mol. Biol. Evol..

[17] Sow R.S., Thiongane P.I., Tchamutchina L. (1988). Bilan de cinq années d’études des moutons Peul et Touabire au Centre de Recherche Zootechnique de Dahra-Djoloff. Rev. Sénégalaise Des. Rech. Agric. Et Halieut..

[18] Jeanpierre M. (1987). A rapid method for the purification of DNA from blood. Nucleic Acids Res.

[19] Sempéré G., Moazami-Goudarzi K., Eggen A., Laloë D., Gautier M., Flori L. (2015). WIDDE: A Web-Interfaced next generation database for genetic diversity exploration, with a first application in cattle. BMC Genom..

[20] Kijas J.W., Lenstra J.A., Hayes B., Boitard S., Porto Neto L.R., San Cristobal M., Servin B., McCulloch R., Whan V., Gietzen K. (2012). Genome-Wide Analysis of the World’s Sheep Breeds Reveals High Levels of Historic Mixture and Strong Recent Selection. PLoS Biol..

[21] Ciani E., Lasagna E., D’Andrea M., Alloggio I., Marroni F., Ceccobelli S., Delgado Bermejo J.V., Sarti F.M., Kijas J., Lenstra J.A. (2015). Merino and Merino-derived sheep breeds: A genome-wide intercontinental study. Genet. Sel. Evol..

[22] Young E.A., Kijas J.W., Mcculloch R., Scobie D.R., Mcrae K.M., Pickering N.K., Dodds K.G., Mcewan J.C. (2011). Arapawa: A novel New Zealand sheep breed of distinct origin. Proc. New Zealand Soc. Anim. Prod..

[23] Patterson N., Price A.L., Reich D. (2006). Population Structure and Eigenanalysis. PLoS Genet..

[24] Chessel D., Dufour A., Thioulouse J. (2004). The ade4 package-I-One-table methods. R News.

[25] Alexander D.H., Novembre J., Lange K. (2009). Fast model-based estimation of ancestry in unrelated individuals. Genome Res..

[26] Nei M. (1978). Estimation of average heterozygosity and genetic distance from a small number of individuals. Genetics.

[27] Weir B.S., Cockerham C.C. (1984). Estimating F-Statistics for the Analysis of Population Structure. Evolution.

[28] De Meeûs T. (2012). Initiation à la Génétique des Populations Naturelles: Application aux Parasites et à Leurs Vecteurs.

[29] Paradis E., Claude J., Strimmer K. (2004). APE: Analyses of Phylogenetics and Evolution in R language. Bioinformatics.

[30] Beynon S.E., Slavov G.T., Farré M., Sunduimijid B., Waddams K., Davies B., Haresign W., Kijas J., MacLeod I.M., Newbold C.J. (2015). Population structure and history of the Welsh sheep breeds determined by whole genome genotyping. BMC Genet..

[31] Molotsi A.H., Taylor J.F., Cloete S.W.P., Muchadeyi F., Decker J.E., Whitacre L.K., Sandenbergh L., Dzama K. (2017). Genetic diversity and population structure of South African smallholder farmer sheep breeds determined using the OvineSNP50 beadchip. Trop. Anim. Health Prod..

[32] Spangler G.L., Rosen B.D., Ilori M.B., Hanotte O., Kim E.-S., Sonstegard T.S., Burke J.M., Morgan J.L.M., Notter D.R., Van Tassell C.P. (2017). Whole genome structural analysis of Caribbean hair sheep reveals quantitative link to West African ancestry. PLoS ONE.

[33] Hoda A., Hykaj G., Sena L., Delia E. (2011). Population structure in three Albanian sheep breeds using 36 single nucleotide polymorphisms. Acta Agric. Scand. Sect. A Anim. Sci..

[34] Álvarez I., Traoré A., Tamboura H.H., Kaboré A., Royo L.J., Fernández I., Ouédraogo-Sanou G., Sawadogo L., Goyache F. (2009). Microsatellite Analysis Characterizes Burkina Faso as a Genetic Contact Zone Between Sahelian and Djallonké Sheep. Anim. Biotechnol..

[35] Wafula P., Jianlin H., Sangare N., Sowe J., Coly R., Diallo B., Hanotte O. (2005). Genetic characterization of West African djallonke sheep using microsatellite markers. Turin Role Biotechnol..

[36] Dayo G.-K., Houaga I., Somda M.B., Linguelegue A., Ira M., Konkobo M., Djassi B., Gomes J., Sangare M., Cassama B. (2022). Morphological and microsatellite DNA diversity of Djallonké sheep in Guinea-Bissau. BMC Genom. Data.

[37] Deniskova T.E., Dotsev A.V., Selionova M.I., Kunz E., Medugorac I., Reyer H., Wimmers K., Barbato M., Traspov A.A., Brem G. (2018). Population structure and genetic diversity of 25 Russian sheep breeds based on whole-genome genotyping. Genet. Sel. Evol..

[38] MacHugh D.E., Shriver M.D., Loftus R.T., Cunningham P., Bradley D.G. (1997). Microsatellite DNA variation and the evolution, domestication and phylogeography of taurine and zebu cattle (*Bos taurus* and *Bos indicus*). Genetics.

[39] Missohou A., Adakal E.H. Situation actuelle et perspectives d’une gestion durable des ressources génétiques bovines d’Afrique de l’Ouest. Proceedings of the Colloque sur le Développement Durable: Leçons et Perspectives.

[40] Rege J.E.O. (1999). The state of African cattle genetic resources I. Classification framework and identification of threatened and extinct breeds. Anim. Genet. Resour. Inf..

[41] Cesaro J.-D., Magrin G., Minot O. (2010). Atlas de L’élevage au Sénégal.

[42] Sandenbergh L., Cloete S.W.P., Roodt-Wilding R., Snyman M.A., Merwe A.E.v.d. Genetic diversity and population structure of four South African sheep breeds. Proceedings of the Association for the Advancement of Animal Breeding and Genetics.

[43] Doutressoulle G. (1947). L’élevage en Afrique Occidentale Française.

[44] Epstein H. (1971). The Origin of the Domesticated Animals of Africa.

[45] Cañón J., García D., García-Atance M.A., Obexer-Ruff G., Lenstra J.A., Ajmone-Marsan P., Dunner S. (2006). Geographical partitioning of goat diversity in Europe and the Middle East. Anim. Genet..

[46] Peter C., Bruford M., Perez T., Dalamitra S., Hewitt G., Erhardt G., Consortium T.E. (2007). Genetic diversity and subdivision of 57 European and Middle-Eastern sheep breeds. Anim. Genet..

[47] Gifford-Gonzalez D., Hanotte O. (2011). Domesticating Animals in Africa: Implications of Genetic and Archaeological Findings. J. World Prehistory.

